# Intrinsically photosensitive retinal ganglion cells may disrupt the effects of visual cycle suppression in central serous chorioretinopathy

**DOI:** 10.5935/0004-2749.2022-0125

**Published:** 2025-08-21

**Authors:** Manish Jain

**Affiliations:** 1 Department of Ophthalmology, Veer Chandra Singh Garhwali, Government Institute of Medical Sciences & Research, Srinagar 246174, Uttarakhand, India

Dear Editor,

I have read with interest the article on the effect of covering the sick eye for 48 h in central serous retinopathy (CSCR) by Zhao et al.^(1)^. The authors have provided objective evidence toward improvement in visual acuity, macular thickness, and the amplitude in multifocal electroretinogram. They suggest that the interruption of the visual cycle can suppress the phototoxic stress and thereby allow the retino-choroid to recover. Since, in addition to the classical visual cycle, photo-responsive mechanisms have been elucidated, the potential effect of visual cycle suppression may have been inadequate. Conversely, if the two systems are synchronized appropriately, the visual recovery in acute central serous retinopathy (CSCR) may further hasten. We then rec apitulated the role of melanopsin containing intrinsically photosensitive retinal ganglion cells (ip-RGC) in the circadian rhythm^(2)^.

The hypothalamus-pituitary-adrenal (HPA) axis is implicated in the role of endogenous Cushing syndrome and stress in CSCR. The diurnal variation in the levels of endogenous cortisol and other hormones is regulated by the circadian rhythm. However, a hypothalamic master clock, and, more precisely, the suprachiasmatic nucleus (SCN) rely on visual cues on recalibrating these levels. Of the 3 major afferent inputs, the most pertinent in the current context is the one coming via the retinohypothalamic tract (RTT) through which photopic information received by rod/cone and ip-RGC is sent to the SCN^(3,4)^. The other two projections are the median raphe nucleus and the geniculohypothalamic tract (GHT); these three projections employ glutamate, serotonin, and neuropeptide Y, respectively.

Melanopsin in the ip-RGC controls the production of melatonin by the pineal gland. Serotonin’s conversion to melatonin is regulated by dopamine and both serotonergic and dopaminergic activities have been previously suggested to be linked to CSCR. Normally, serotonin prevails under the photopic conditions, while melatonin takes over under the scotopic conditions^(2)^. On exposure to blue-enriched white light, ip-RGC efficiently suppress the melatonin at night. Thus, blue light can be seen as the most important zeitgeber. It also augments the production of reactive oxygen species and the consequent oxidative damage, leading to the build-up of lipofuscin in the retinal pigment epithelium. Effectively, the HPA axis receives vital inputs from ip-RGC via the SCN. Recent evidence suggests that circadian-related heteromerization of adrenergic and dopaminergic receptors modulates melatonin in the pineal body, resulting in complex interactions between catecholamines and melatonin^(4)^.

In anatomical terms, SCN is a part of the complete neural loop that includes the paraventricular nucleus, intermediolateral tract of the spinal cord, and, finally, the superior cervical ganglion (SCG)^(3)^. The choroid receives its sympathetic innervation from the SCG. This, along with the parasympathetic and the intrinsic choroidal neuronal influences, determines the choroidal blood flow (ChBF). Understandably, the superior cervical gangliectomy reduces the choroidal thickness in animal models^(5)^.

The SCN is paired nuclei with bilateral connections, implying that unilateral inputs can have bilateral effects on the ocular physiology, including the ChBF. While the authors successfully demonstrated improvement in CSCR by covering the sick eye, the uncovered eye continued to influence the ChBF inappropriately.

Understandably, not all CSCR patients have a primary circadian rhythm disturbance; however, apart from shift workers, airline crew members who fly across the time zones, modern life necessitates many people to work late nights with ubiquitous blue light-emitting devices. Covering both eyes to enhance recovery is not feasible. Nevertheless, based on the observations of Zhao et al. and the above-mentioned discussion, it is suggested that patients with ambient predispositions to circadian rhythm disturbances must be counseled to avoid undue photopic stimulation of the ip-RGC.

CSCR is bilateral disease, as evidenced by frequent changes observed in the “unaffected eye”, such as the pachychoroid. It exerts multiple central/peripheral influences. Thus, neuro-biologically, it must be addressed by managing the retinohypothalamic tract (RHT) inputs, at least in a subgroup of patients who have such predispositions. Furthermore, the indirect influences of the SCN on the endocrinal system imply that other patients with psychological stress and endogenous corticosteroids surge may also benefit from such advice. Patients with bilateral manifestations of the disease may be advised to cover each eye on alternate days. Of course, further research is warranted to link the advances in basic neurobiology to clinical events in CSCR.
